# Book Review on "Modern Uterine Cytopathology: Moving to the Molecular Smear" by Alexander Meisels MD and Carol Morin, MSc, PhD

**DOI:** 10.1186/1742-6413-4-8

**Published:** 2007-04-13

**Authors:** Barbara Mahoney

**Affiliations:** 1 Medical Laboratories of Windsor, Ontario, Canada – 1428 Ouellette Ave., Suite 201, Windsor, ON, N8X 1K4. USA

The authors have designed this book to be a practical reference guide for working cytopathology professionals. It is meant to bridge the gap between cytomorphology as practiced for the last half century with the new molecular biology and molecular technologies.

Since Drs. Meisels and Fortin published their pivotal paper on "Condylomatous Lesions of the Cervix and Vagina I: cytologic patterns". Acta Cytologica. 20: 505-9, 1976 the field of gynecologic cytopathology has grown exponentially. This discovery that HPV has premalignant and malignant potential is the most important event in Cytopathology since the introduction of Cytology as a diagnostic tool by Dr. George N. Papanicolaou. It has spawned an enormous amount of new research on the morphogenesis and carcinogenesis of cervical cancer in women.

Chapter One,"Cytopathology of the Uterus: Historical Perspectives" by Dr. Bernard Naylor, traces the earliest publications of cellular biologists in the 1800's, through the major cytological writings of the 20th and the 21^st ^century. This chapter is a scholarly, detailed account of the early investigators and their works including: Dr. George N. Papanicolaou, Dr. Aurel Babes, Dr. Herbert Traut, Dr. J. Ernest Ayre; just to name a few. Dr. Naylor notes the contributions of Dr. Alexander Meisels and his colleagues with their description of the Human Papillomavirus infection in the koilocyte and its link to cervical cancer. Dr. Naylor's discussion continues with screening programs for cervical cancer, the pap Smear Exposé of 1987 and all of its consequences, the beginnings of The Bethesda system (TBS) Reporting System for Gynecologic specimens, the advent of the new Liquid-Based preparations for Cervical Cytology Screening and the development of Automated Screening Technologies.

From chapters 2 through 7 Cytopathology of Squamous lesions, Glandular Lesions, Rare Lesions of the Uterus are discussed as well as infections and benign lesions and Hormonal Cytology. These topics are all discussed concisely and flawlessly.

Chapters 8 through 18 cover Epidemiology, Human Papillomavirus, and cervical Carcinogenesis, Biomarkers in Cervical Cancer, Quality Assurance, Molecular and Serologic Diagnosis of HPV Infections;

There are extensive technical tips and procedural notes given as well as listings of the Guidelines for the Management of Women with Cytological Abnormalities.

Chapter 16, "A Brief history of Cytology Automation" is a very balanced, unbiased account of events leading up to the present climate of liquid-based Pap smears being the norm rather than the exception. Author Dr. Dorothy Rosenthal discusses all aspects of this subject including the ramifications of "placing automated instruments on the witness stand" in a legal case.

This textbook, written by Dr. Alexander Meisels and his co-author Dr. Carol Morin, is a complete, all inclusive, comprehensive reference manual of relevant issues, diagnostic entities, methodologies and technologies in uterine cytopathology. This book contains contributions from eleven other Cytopathology experts from North America and Europe. Concise information coupled with copious relevant references and exemplary photomicrographs when appropriate. The aesthetic qualities of this book begin with the front cover logo of the three koilocytes surrounded by the symbols representing the organizational structure of the papillomavirus genome for HPV – 16. This cutting edge desktop reference manual containing outstanding photomicrographs should be included in personal and professional libraries.

All of the figures and tables are beautifully designed. All illustrations and photomicrographs are aligned with the explanatory text. The photographic image numbers are highlighted in various colors, and also in the corresponding text, giving them prominence in the text, thus facilitating reading. The graphics of the tables are color coded which highlights the corresponding text.

"Modern Uterine Cytopathology: Moving to the Molecular Smear" bridges the gap from the early years of cervical cytopathology through the present transitional period of molecular biology and new cytopathology and molecular technologies. The book is unparalleled in its scope and theme.

Dr. Alexander Meisels is an internationally known pioneer in Cytopathology; an extraordinary researcher, tireless educator and superb diagnostician who has lead us down an exciting path in the last thirty years since his discovery that Human Papillomavirus is the etiologic agent in cervical cancer.

It is only fitting then, that he and his colleagues have written this quintessential reference textbook that will lead the Cytopathology community through our present transitional period into the exciting future of uterine cytopathology.

"Modern Uterine Cytopathology : Moving to the Molecular Smear" is destined to become a perennial classic in Cytopahology reference publications.

I highly recommend this book.

Barbara Mahoney M.Sc., CT, (ASCP), ( IAC), (CSMLS).

Medical Laboratories of Windsor, Windsor, Ontario, Canada.

